# The complete chloroplast genome of *Physochlaina physaloides* (Solanaceae), an important medicinal plant

**DOI:** 10.1080/23802359.2019.1674730

**Published:** 2019-10-09

**Authors:** Ling Tong, Yi-Xuan Zhu, Feng-Wei Lei, Xue-Li Shen, Xian-Yun Mu

**Affiliations:** Laboratory of Systematic Evolution and Biogeography of Woody Plants, College of Nature Conservation, Beijing Forestry University, Beijing, P. R. China

**Keywords:** *Physochlaina physaloides*, complete chloroplast genome, Hyoscyameae, Solanaceae, medicinal plant

## Abstract

*Physochlaina* is an important perennial herbaceous genus with significant medicinal value, while the phylogeny of *Physochlaina* and tribe Hyoscyameae is not well resolved yet. In this study, we report the complete chloroplast genome sequences of *Ph. physaloides*, its complete chloroplast genome is 156,413 bp in length, which is a typical quadripartite structure that includes a large single-copy region of 86,659 bp, a small single-copy region of 18,012 bp, and its GC content was 37.7%. A total of 132 genes were identified, including 87 protein-coding genes, 37 tRNA genes, and 8 rRNA genes. Furthermore, a phylogenetic tree of the tribe Hyoscyameae was constructed based the complete chloroplast genome sequence, and a new topology of the tribe was obtained. This study provides valuable genetic information for the conservation and utilization of *Ph. physaloides* and also provide the potential for better understanding of the phylogeny of Hyoscyameae and Solanaceae.

*Physochlaina* is a perennial herbaceous genus of the tribe Hyoscyameae in Solanaceae with significant medicinal value, such as anti-inflammatory, relieving muscular spasm and pain, and haemostasis (Hong et al. 2018). Furthermore, several chemical compounds in *Physochlaina* are also found to be hallucinogenic and poisonous. Among the 11 species in the genus, *Ph. physaloides* is the only one that distributes from Northwest China to Northeast China and Siberia and is widely used as a traditional medicine in Inner Mongolia. Except its value in medicine, we know little about the phylogeny of *Ph. physaloides*. Although the monophyly of the tribe Hyoscyameae is recognized, phylogenetic relationship among genera in the tribe is not well resolved and need further investigation (Tu et al. 2010; Särkinen et al. 2013; Sanchez-Puerta and Abbona 2014). In this study, we reassembled and annotated the chloroplast genome of *Ph. physaloides*, with the aim to provide genetic information for its conservation and utilization and help further understanding the phylogeny of Hyoscyameae.

The fresh leaves of *Ph. physaloides* were collected from Yudu Mountain in Yanqing District, Beijing City, China (N40°33′89.10″, E115°51′35.89″). Voucher specimen (collector and collection number: *Xian-Yun Mu 4445*) is deposited in the herbarium of Beijing Forestry University (BJFC). Genomic DNA was extracted and sequenced by next-generation sequencing method on Illumina Hiseq platform. In total, 13 Gb of 150-bp clean reads were generated and used for chloroplast genome assembly which was performed on Geneious 11.1.4 software (Kearse et al. 2012) with *Ph. orientalis* (Gandini et al. 2019) as a reference, followed by annotation using Plann (Huang and Cronk 2015) and further verification by Geneious 11.1.4 software. Chloroplast genome of *Ph. physaloides* is submitted to NCBI with the accession number MN262642.

The complete chloroplast genome of *Ph. physaloides* was 156,413 bp in length. The genome has a typical quadripartite structure, including a large single-copy region of 86,659 bp, a small single-copy region of 18,012 bp, and two inverted repeat (IR) regions of 25,871 bp for each. Overall, the GC content was 37.7%. A total of 132 genes were identified in the chloroplast genome of *Ph. physaloides*, including 87 protein-coding genes, 37 tRNA genes, and 8 rRNA genes.

Phylogenetic analysis of the tribe Hyoscyameae was reconstructed based on representatives of six genera, and *Nicotiana tabacum* as outgroup. Sequences were aligned using MAFFT (Katoh et al. 2017), and a maximum likelihood tree with bootstrap value was constructed by IQ-TREE (Hoang et al. 2018) (Figure 1). Contrary to several chloroplast genome markers used in previous studies, full length of chloroplast genome sequences were analyzed for phylogeny in this study, and a new topology was presented. *Physochlaina physaloides* was resolved as the sister of *Ph. orientalis* with strong support values. However, the closet sister of *Przewalskia* that demonstrated as *Physochlaina* in previous studies is substituted by *Scopolia* with poor support in this study. Phylogenetic position of *Hyoscyamus* also varies among the current and previous studies. Hence, it is of great value to make further investigation about the phylogeny of Hyoscyameae and Solanaceae based on whole chloroplast genome sequence data in the future.

**Figure 1.**
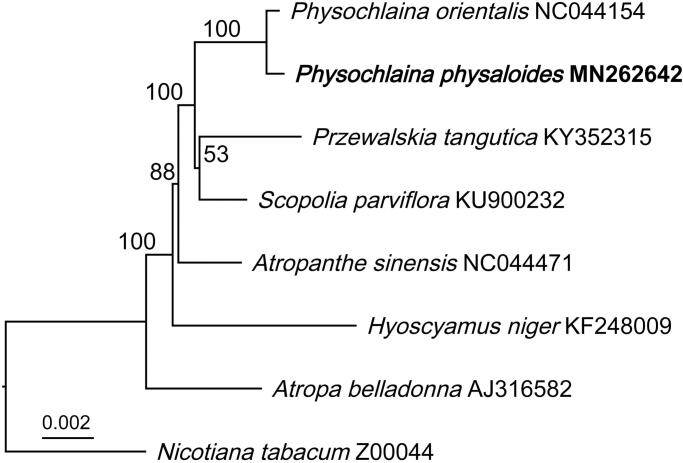
A maximum-likelihood tree of Hyoscyamee (Solanaceae) based on complete chloroplast genome sequence data and *Nicotiana tabacum* as an outgroup. The position of *Physochlaina physaloides* is shown in bold. Bootstrap values of maximum likelihood analysis are presented above nodes.

## References

[CIT0001] Gandini CL, Garcia LE, Abbona CC, Sanchez-Puerta MV 2019 The complete organelle genomes of *Physochlaina orientalis*: insights into short sequence repeats across seed plant mitochondrial genomes. Mol Phylogenet Evol. 137:274–284. 31112782 10.1016/j.ympev.2019.05.012

[CIT0002] Hoang DT, Chernomor O, von Haeseler A, Minh BQ, Vinh LS 2018 UFBoot2: improving the ultrafast bootstrap approximation. Mol Biol Evol. 35:518–522. 29077904 10.1093/molbev/msx281 PMC5850222

[CIT0003] Hong Y, Wang QH, Hao JS, Buhebateer B 2018 Isolation and identification of chemical constituents from *Physochlaina physaloides*. Chin J Med Chem. 28:232–236.

[CIT0004] Huang DI, Cronk QC 2015 Plann: a command-line application for annotating plastome sequences. Appl Plant Sci. 3:1500026. 10.3732/apps.1500026 PMC4542940 26312193

[CIT0005] Katoh K, Rozewicki J, Yamada KD 2017 MAFFT online service: multiple sequence alignment, interactive sequence choice and visualization. Brief Bioinform. In press. 10.1093/bib/bbx108 PMC6781576 28968734

[CIT0006] Kearse M, Moir R, Wilson A, Stones-Havas S, Cheung M, Sturrock S, Buxton S, Cooper A, Markowitz S, Duran C, et al. 2012 Geneious Basic: an integrated and extendable desktop software platform for the organization and analysis of sequence data. Bioinformatics. 28:1647–1649. 22543367 10.1093/bioinformatics/bts199 PMC3371832

[CIT0007] Sanchez-Puerta MV, Abbona CC 2014 The chloroplast genome of *Hyoscyamus niger* and a phylogenetic study of the tribe Hyoscyameae (Solanaceae). PLoS One. 9:e98353. 24851862 10.1371/journal.pone.0098353 PMC4031233

[CIT0008] Särkinen T, Bohs L, Olmstead RG, Knapp S 2013 A phylogenetic framework for evolutionary study of the nightshades (Solanaceae): a dated 1000-tip tree. BMC Evol Biol. 13:214. 24283922 10.1186/1471-2148-13-214 PMC3850475

[CIT0009] Tu T, Volis S, Dillon MO, Sun H, Wen J 2010 Dispersals of Hyoscyameae and Mandragoreae (Solanaceae) from the New World to Eurasia in the early Miocene and their biogeographic diversification within Eurasia. Mol Phylogenet Evol. 57:1226–1237. 20858548 10.1016/j.ympev.2010.09.007

